# Surface Conditions after LASER Shock Peening of Steel and Aluminum Alloys Using Ultrafast Laser Pulses

**DOI:** 10.3390/ma16206769

**Published:** 2023-10-19

**Authors:** Jan Schubnell, Eva-Regine Carl, Ardeshir Sarmast, Manuel Hinterstein, Johannes Preußner, Marco Seifert, Christoph Kaufmann, Peter Rußbüldt, Jan Schulte

**Affiliations:** 1 Fraunhofer Institute for Mechancis of Materials IWM, Woehlerstr. 11, 79109 Freiburg, Germany; 2 Fraunhofer Institute for Material and Beam Technology IWS, Winterbergstr. 18, 01277 Dresden, Germany; 3 Fraunhofer Institute for Laser Technology, Steinbachstr. 15, 52074 Aachen, Germany

**Keywords:** laser shock peening, shot peening, residual stresses, surface roughness, microstructure, aluminum alloys, steel

## Abstract

Laser shock peening (LSP) is a mechanical surface treatment process to modify near-surface material properties. Compared to conventional shot peening (SP) the process parameters can be finely adjusted with greater precision and a higher penetration depth of compressive residual stresses could be reached. However, high process times of LSP leads to high production costs. In this study, ultrafast LSP (U-LSP) with an ultrafast laser source (pulse time in the picosecond range) was applied on specimens made of X5CrNiCu15-5 and AlZnMgCu1.5. The surface characteristics (surface roughness) and surface-near properties (microstructure, residual stresses, and phase composition) were compared to the as-delivered condition, to conventional laser shock peening (C-LSP), and to SP, whereas metallographic analyses and X-ray and synchrotron radiation techniques were used. The process time was significantly lower via U-LSP compared to C-LSP. For X5CrNiCu15-5, no significant compressive residual stresses were induced via U-LSP. However, for AlZnMgCu1.5, similar compressive residual stresses were reached via C-LSP and U-LSP; however, with a lower penetration depth. A change in the phase portions in the surface layer of X5CrNiCu15-5 after C-LSP compared to SP were determined.

## 1. Introduction

**Figure 1:**
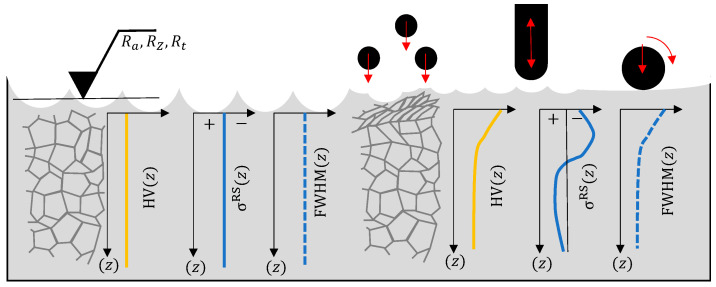
Surface layer characteristics influenced by surface modification processes, schematically.

More than 80% of the failure of parts and components of metallic materials are caused by wear, corrosion, or fatigue [1]. Most of these failures originate at the material’s surface. The most important features to describe the characteristics of the surface are topography measurements including, e.g., roughness $R_{Z}$, $R_{a}$, $R_{t}$ and waviness. Characteristic features of the surface-near area include, for example, the hardness HV(z) and full width half maximum FWHM(z), the microstructure, and the residual stress state $\sigma^{RS}(z)$ [2], as illustrated in Figure 1. Mechanical surface treatment methods like shot peening, deep rolling, or hammer peening are designed to modify these characteristics in a beneficial way. Shot peening (SP) is a process which is established in the industry and leads to work hardening and to the induction of compressive residual stresses in the surface-near area. However, SP is a stochastic process that usually leads to an increase in the surface roughness and needs a strict quality control, e.g., via Almen strip or visual or non-destructive crack monitoring for sensitive and high-quality parts like turbine components, leaf springs [3], or landing gears [4].

Conventional Laser Shock Peening (LSP) exposes the surface of a workpiece to laser pulses with a pulse duration in the nanosecond (ns) range and is used for the mechanical treatment of surface-near regions [2]. LSP in comparison to SP has several advantages [1]: the process parameters can be precisely controlled during the process, the induction of compressive residual stresses in deeper layers, a high process efficiency, and a clean working environment. Especially for high-end parts with high requirements regarding the surface integrity (aircraft and aerospace), the application of LSP seems to be more beneficial than conventional SP [5]. Multiple investigations have shown the beneficial effect of LSP on typical Ti-, Al-, Fe-, and Ni-alloys like Ti-6AL-4V [6], EN-AW 2024 [7], EN-AW 7075 [8], [9], 15-5 PH [3], and Inconel 718 [10]. However, compared to SP, investments and production costs (process time) are much higher for the application of LSP. Especially in mass production, the high process times by LSP make this process currently not attractive for many industrial applications except for high-end parts.

For these reasons, the aim of the current study is investigating the potential of process time decrease in LSP while generating sufficient surface characteristics. Therefore, a newly developed laser system by the Fraunhofer CAPS Cluster (Cluster of Advanced Photon Sources) was used. The system is able to decrease the pulse time in the nanosecond range (conventional LSP, C-LSP) and in the picosecond range (ultrafast LSP, U-LSP). Currently the limiting factor regarding U-LSP is the laser pulse energy of the laser source. As the benchmark for the U-LSP process, a specimen made of two commonly used Al- und Fe-alloys were treated with C-LSP and SP, see Section 2. To quantify the surface characteristics, X-ray and synchrotron analysis techniques were used, see Section 3. The determined surface characteristics are summarized in Section 4. In Section 5, conclusions regarding the aim of this study are made.

## 2. Materials and Surface Treatment Methods

### 2.1. Investigated Materials and Specimen

Two materials were used for this investigation: a precipitation-hardened martensitic steel X5CrNiCu15-5 (15-5 PH, 1.4545.4) and a high-strength aluminum alloy AlZnMgCu1.5 (AAW-7075, 3.4365) in the T7351/651 condition according to DIN EN 573-3 [11]. The X5CrNiCu15-5 steel was annealed and grit-blasted in the as-delivered condition (AD). The AlZnMgCu1.5 alloy was milled in the AD condition. Both materials represent typical industrial applications in mechanical engineering or aerospace industry and have a wide range of mechanical properties. The chemical compositions of both materials are summarized in Table 1. The mechanical properties are given in Table 2. Specimens with the dimensions of 140 × 40 × 8 mm^3^ were manufactured from both materials. For all subsequent treatment methods, a surface of 60 × 40 mm^2^ was treated.

### 2.2. Shot Peening (SP)

As the reference process (benchmark), conventional shot peening (SP) was performed in the laboratory of the company OSK Kiefer. Ceramic shots type B20 with a diameter between 600 and 850 µm were used. The Almen intensity of the process was 0.24 mmA with a coverage of 125% at a time of 30 s. The ceramic shots were chosen according to the current industrial applications for these materials. The peening time of 30 s was used according to SAE J442 to maximize the induced compressive residual stresses. 

### 2.3. Conventional Laser Shock Peening (C-LSP)

**Figure 2:**
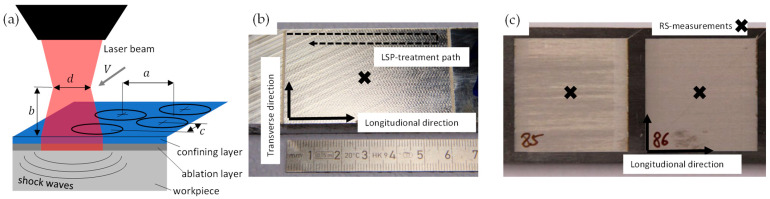
(**a**) Illustration of LSP process parameters, (**b**) C-LSP treated specimen, and (**c**) U-LSP treated specimen.

As the second reference process, conventional laser shock peening (C-LSP) was performed at the laboratory of the Fraunhofer Institute of Material and Beam Technology IWS. As an ablation/confining layer, plastic foil and water were used. The defocusing distance, see Figure 2a, was zero. The number of shots (number of treatment runs) varied with 1, 2, and 4. The defocusing distance b was 0 mm and the stepover distance a was 1.0 mm.

The process parameters are summarized in Table 3 and Table 4. The process parameters are illustrated in Figure 2a. Figure 2b shows the treatment results of the C-LSP process on a specimen made of the AlZnMgCu1.5. For C-LSP and U-LSP, the maximum pulse energy of the laser sources was used.

### 2.4. Ultrafast Laser Shock Peening (U-LSP)

The LSP treatment with an ultrafast laser (pulse time around 1 pico-second) was performed at the laboratory of the Fraunhofer Institute for Laser Technology (ILT) in Aachen. The treatment was performed on the same specimen as SP and C-LSP but within an area of 35 × 35 mm. The used process parameters are summarized in Table 3 and Table 4. Figure 2c shows the treatment results of the C-LSP process on a specimen made of AlZnMgCu1.5. For the investigation of process parameters, the following parameters are varied: feed rate, confining layer, stepover distance and coverage, defocusing distance, and the number of shots. For the number of shots of 2, 3, or 4, an offset c of 0.05 mm, 0.025 mm and 0.075 mm were used. 

## 3. Residual Stress and Phase Analysis

### 3.1. Metallography and Hardness

For the quantification of the influence of the surface treatment methods on microstructure and hardness (work hardening effect), hardness measurements were performed and microstructural images were taken. Hardness HV 0.01 according to ISO 6507 [12] was analyzed with a hardness testing device Qness 60 A+ EVO (ATM Qness GmbH). Microstructure images were taken with an Optical light microscope type Nikon Eclipse ME 600.

### 3.2. Surface Roughness 

To quantify the effect of the U-LSP treatment compared to other methods, surface roughness values $R_{a}$ and $R_{z}$ according to DIN 4768:1990-05 [13] were determined. For this, the tactile measurement device Hommel T8000 was used according to DIN EN ISO 3274:1998–04 [14] within a distance of 4.8 mm in a transverse direction. Roughness and waviness were separated according to DIN EN ISO 4287:2010–07 [15].

### 3.3. Residual Stress Analysis 

Residual stresses are mainly measured using diffraction techniques. There are four main diffraction methods that are widely used for the measurement of the residual stresses, the sin^2^ψ method, and the cosα method, which can be applied with laboratory diffractometers as well as synchrotron and neutron diffraction methods [16,17].

#### 3.3.1. Sin^2^ψ Method

**Figure 3:**
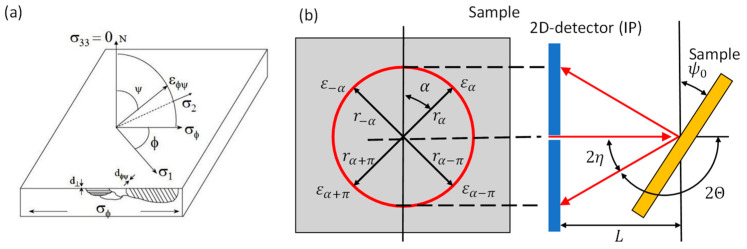
Principle of the XRD analysis via the sinψ*^2^* method (**a**) and the cosα method (**b**).

Residual stresses can be determined via the X-ray diffraction technique. Residual stresses change the lattice spacing of an unstrained crystal. A decrease or increase in the lattice spacing appears as the angular shift in position of the diffraction line according to Bragg’s law. With the position of the diffraction line 2θ at the tilt angle ψ, the residual stresses are calculated according to the slope of the sin2ψ-2θ diagram (Equation (1)). For polycrystalline materials, the sin^2^ψ method [18] has been widely used. Corresponding formulations for the calculation of the strain and stress in a given direction are as below [17,19]:

(1) $$\frac{d_{\psi }-d_{0}}{d_{0}}=\frac{1+\nu }{E}\sigma_{\phi }{Sin}^{2}\psi -\frac{\upsilon }{E}\left(\sigma_{11}+\sigma_{22}\right)$$

(2) $$\sigma_{\phi }=\frac{E}{(1+\upsilon ){Sin}^{2}\psi }\left(\frac{d_{\psi }-d_{n}}{d_{n}}\right)$$

where *E* is the elastic modulus, ν is the Poisson’s ratio, ψ is the tilt angle, d_ψ_ is the lattice spacing at the tilt angle ψ, and $d_{0}$ is the lattice spacing of a stress-free sample. Figure 3a shows a schematic representation of the diffraction planes for the evaluation with the sin^2^ψ method [19]. In equation (2), $d_{0}$ is replaced with $d_{n},$ which is the lattice spacing of ψ = 0. It is the main advantage of the sin^2^ψ method that we can replace the strain-free lattice spacing ($d_{0}$), which is difficult to obtain, with $d_{\psi }\;$= 0 without a significant error as the $d_{0}$ value is a multiplier to the slope of the sin^2^ψ-2θ curve [17]. Zero-dimensional or one-dimensional detectors are usually used. For the aim of this study, a D8 Discover laboratory X-ray diffractometer was used. A one dimensional Lynxeye XE-T detector was used for the data collection. 

#### 3.3.2. cos*α* Method

The stress analysis via the cosα method according to Taira et al. [20] is based on a strain evaluation over the complete Debye–Scherrer ring based on a 2D-detector (digital image plate, IP). The reliability of this method in combination with the 2D-detector compared to the commonly used sinψ^2^ method [18] was shown by Sasaki et al. [21,22,23] for austenitic steels ({311} lattice plane) as well by [24] for martensitic steels ({211} lattice plane). Furthermore, Sarmast et al. [25] showed a high agreement between the RS measurements acquired with the cosα and sinψ^2^ method for a wide range of materials and the material conditions if shear stresses are not present or low. It is assumed that shear stresses influence the measurement result for welded joints.

For the RS evaluation according to the cosα method, the strain in circumferential direction is measured via a shift of the diffraction angle $\theta_{\alpha }$ or radius $r_{\alpha }$ depending on the α position on the detector, as shown in Figure 3b. The detector distance to the specimen L and tilt angle $\psi_{0}$ was constant during the measurement. A strain parameter $\varepsilon_{a1}$ is defined based on the four strains from α = 0° to 90°. The stress $\sigma_{\phi }$ is calculated according to Equations (3) and (4). A detailed description of the method was published by [26].

(3) $$\sigma_{\phi }=-\frac{E}{1+\upsilon }\frac{1}{2\sin\eta \sin2\psi_{0}}\frac{\delta \varepsilon_{a1}}{\delta \cos\alpha }$$

(4) $$\mathrm{with}\;\varepsilon_{\alpha 1}=\left[\left(\varepsilon_{\alpha }-\varepsilon_{\pi +\alpha }\right)+(\varepsilon_{-\alpha }-\varepsilon_{\pi -\alpha })\right]/2$$

For the XRD analysis via the cosα method, a diffractometer type Pulstec µ-360 was used. The diffractometer was mounted on an industrial robot type Kuka KR3 R540. The measurement parameters of both methods are summarized in Table 5. The same elastic constants of E = 220 Gpa and v = 0.29 for X5CrNiCu15-5 and of E = 69.74 Gpa and v = 0.348 for AlZnMgCu1.5 was used for both methods.

### 3.4. STRAP Method with Synchrotron Radiation

**Figure 4:**
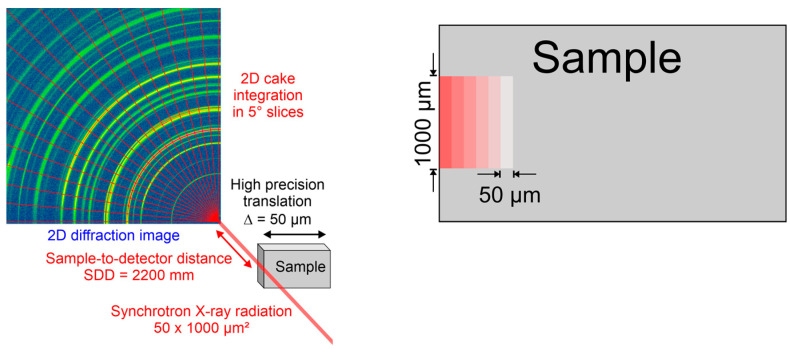
Synchrotron experimental setup and data reduction and scanning setup with 50 µm resolution.

For a depth profile measurement of coexisting phases and residual stresses, the STRAP (Strain, Texture and Rietveld Analysis for Piezoceramics) method [27] was modified and used. The experiments were performed at the P02.1 beamline [28,29] at the *Deutsches Elektronensynchrotron* DESY in Hamburg, Germany. The sample was mounted on an xyz stage with high accuracy (Figure 4). The beam was narrowed to a height of 50 µm and a width of 1 mm. The beam was aligned parallel to the sample surface and scanned from the surface to the bulk of the sample in 50 µm steps (Figure 4). This leads to a depth resolution of 50 µm. The data were collected with a two-dimensional flat panel detector of the XRD 1621 N ES Series (manufacturer PerkinElmer, Waltham, MA, USA) with a sample-to-detector distance of 2200 mm for the highest angular resolution. This leads to the orientation-dependent diffraction data from the perpendicular to the parallel to the sample surface. The data were integrated in 5° slices and analyzed with the software package MAUD v2.7.1 [30]. For the data analysis of the steel samples, a four-phase structure model was used with Austenite and Martensite as well as Hematite and Magnetite for the oxide phases at the surface. A triaxial isotropic stress model was used for modelling the residual stresses in the phases. During the refinements, the residual stresses, phase fractions, lattice parameters, and texture were refined.

## 4. Results

### 4.1. Microstructure and Hardness

**Figure 5:**
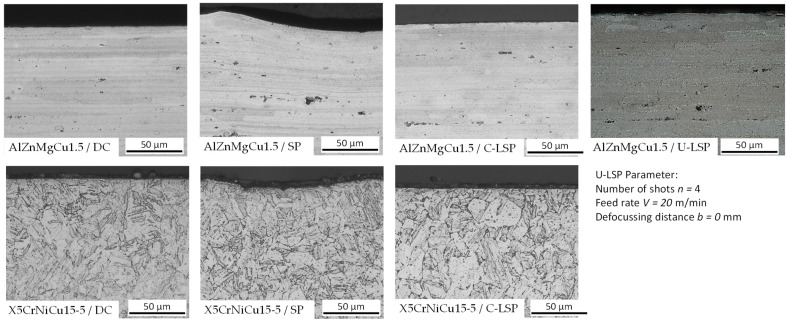
Microstructure in delivered condition (DC), after shot peening (SP), and after conventional Laser Shock Peening (C-LSP).

The surface-near microstructure of X5CrNiCu15-5 and AlZnMgCu1.5 was investigated via an optical light microscope. The microstructure in different conditions is illustrated in Figure 5. No significant influence of Shot Peening or Laser Shock Peening on the grain size or shape could be determined. The micro hardness HV0.01 in a distance of 15 µm from the surface over the first 100 µm also shows no significant changes. The average hardness was 435 HV0.01 for X5CrNiCu15-5 and 203 HV0.01 for AlZnMgCu1.5.

### 4.2. Surface Roughness

**Figure 6:**
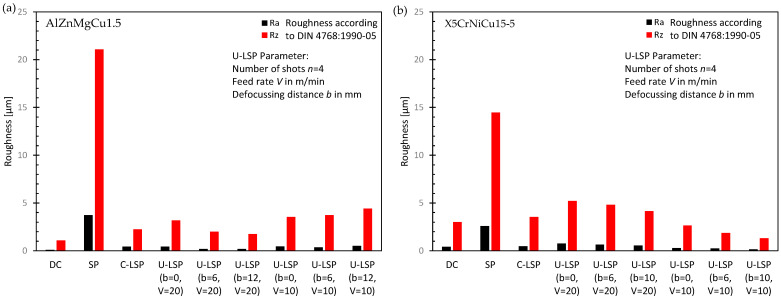
Surface roughness according to DIN 4768 [13] in different conditions for specimens made of AlZnMgCu1.5 (**a**) and X5CrNiCu15-5 (**b**).

The results of the roughness measurements are summarized in Figure 6 (feed rate V is in m/min and defocusing distance b in mm). As shown, shot peening (SP) leads to a significant increase in the surface roughness, while conventional (C-LSP) and ultrafast Laser Shock Peening (U-LSP) lead to roughness values that are in the range of the surface in the delivered condition (DC). For both materials, the roughness values are dependent on the process parameters of the U-LSP process. However, minimum roughness values were determined for AlZnMgCu1.5 with a feed rate of V = 20 m/min, while lower roughness values were determined for the X5CrNiCu15-5 specimen with a feed rate of V = 20 m/min. An increase in the defocusing distance b seems to decrease the surface roughness in both cases.

### 4.3. Residual Stress State

**Figure 7:**
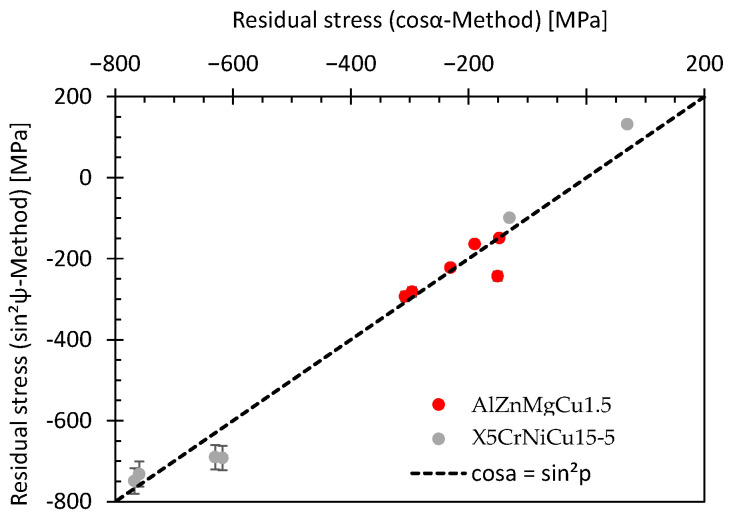
Comparison of the residual stresses determined via the sinψ*^2^* method and the cosα method.

Residual stresses may significantly affect the fatigue and wear resistance of parts and components. Compressive residual stresses in general are classified as beneficial regarding the fatigue behavior under cyclic loading. For this reason, the residual stress state at the surface was determined via X-ray diffraction according to the sinψ^2^ and cosα method, see Section 3.3. As shown in Figure 7, no significant differences were determined between the results of the sinψ^2^ and cosα method. An extensive comparison by Sarmast el al. [23] shows also no significant difference for both methods regarding the residual stresses after SP or LSP. For this reason, further analysis was performed via the cosα method.

To determine the optimum process parameters of the U-LSP for both materials, the parameter feed rate V, stepover distance a, defocusing distance b, number of shots n, and the coating of the surface were varied and compared to the surface after SP and C-LSP and in the DC condition.

**Figure 8:**
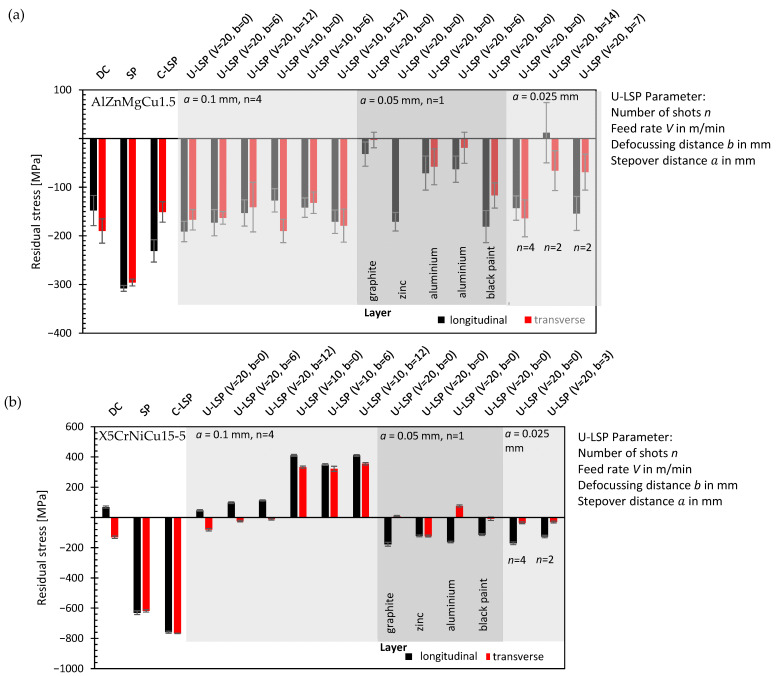
Residual stress state after different U-LSP treatments for AlZnMgCu1.5 (**a**) and X5CrNiCu15-5 (**b**).

The results are summarized in Figure 8. The highest compressive residual stresses were determined after SP for AlZnMgCu1.5. The highest residual stress values after U-LSP are still slightly below the values that could be reached with C-LSP. Regarding the influence of the process parameters for the LSP process, the following trends were determined: graphite and aluminum coatings reduce the induced compressive residual stresses; a change in a from 0.1 mm to 0.05 mm or 0.025 mm does not increase the compressive residual stresses; an increase in b above a value of 12 mm significantly reduces the induced compressive residual stresses; and a decrease in the feed rate V from 20 m/min to 10 m/min does not affect the residual stress state significantly.

For the X5CrNiCu15-5 specimens, the highest compressive residual stresses were determined for the C-LSP process. For the U-LSP process, the determined compressive residual stresses were significantly below the values that were determined after SP or C-LSP. Regarding the influence of the process parameters for the LSP process, the following trends were determined: The U-LSP process with V = 20 m/min does not affect the residual stress state significantly; however, for V = 10 m/min, the tensile residual stresses were determined. Additionally, the application of coatings or a decrease in a seems to increase the compressive residual stresses.

**Figure 9:**
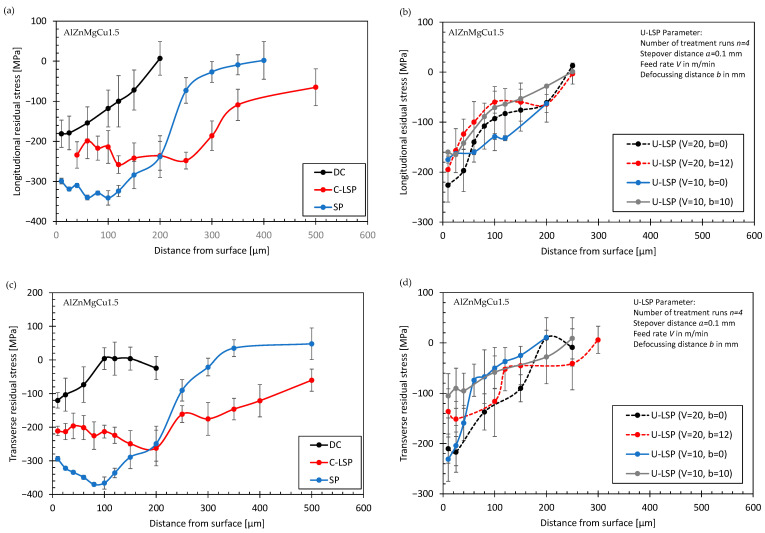
Residual stresses over the specimen thickness for AlZnMgCu1.5 in longitudinal direction for different surface treatment methods (**a**), for different U-LSP parameters (**b**), in transverse direction for different surface treatment methods (**c**), and for different U-LSP parameters (**d**).

Residual stress depth profiles were also determined for the specimens made of AlZnMgCu1.5 to determine the influence of the surface treatment over the specimen thickness. The surface was electro-chemically removed after single measurements. The results are summarized in Figure 9. In the DC condition, a depth of around 100 µm to 160 µm was determined. The highest compressive residual stresses were again determined after SP. However, the penetration depth of the compressive residual stresses are higher after C-LSP. Regarding the influence of the process parameters of U-LSP, the following results were determined: the penetration depths of the compressive residual stress do not significantly change by a variation in V from 10 m/min to 20 m/min or by a change in b from 0 to 12 mm; and slightly higher compressive residual stresses were induced for b = 0 mm compared to b = 12 mm.

### 4.4. Phase Composition

**Figure 10:**
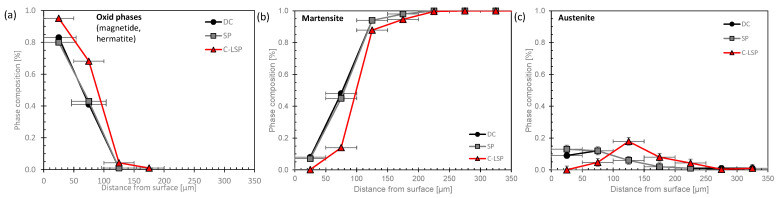
Phase composition of X5CrNiCu15-5 of oxide phases (**a**), martensite phase (**b**), and austenite phase (**c**) in delivered condition (DC), after shot peening (SP), and after conventional laser shock peening (C-LSP).

The phase composition was measured via synchrotron and is given in Figure 10, where the results represent the martensitic microstructure of the 15-5PH steel. Typically, the stainless steel consists out of a martensitic microstructure which is precipitation-hardened. The alloy is often not 100% martensitic and (retained) austenite may be present after the processing route [31]. Indeed, some austenite is present in the surface-near region, see Figure 10c. At the very surface, oxide phases are present. The second measurement point reflects the range between 50 and 100 µm where both oxide phases from the surface and the base material are present.

## 5. Discussion and Conclusions

In this study the surface treatment via ultrafast Laser Shock Peening (U-LSP) with pulse times in the picosecond range and a repetition rate of 400 KHz was compared to conventional Laser Shock Peening (C-LSP) and conventional shot peening (SP) and the delivered condition (DC). The aim of this study was to elucidate if lower treatment times via U-LSP may lead to sufficient surface integrity characteristics and may be an alternative to C-LSP or SP. For the U-LSP process, the parameters were varied to optimize the surface integrity characteristics. For this investigation, the materials X5CrNiCu15-5 and AlZnMgCu1.5 were used. Highest compressive residual stresses were determined for a defocusing distance b of 0 mm. Other parameters except the ablation layer seems to have a minor effect. To quantify the surface condition, microsection analysis, roughness measurements, residual stress analysis, and phase composition analysis were performed. 

Similar compressive residual stresses and a depth of penetration of these stresses of hard-chromed X5CrNiCu15-5 after conventional LSP were determined by Sundar et al. [3]. Thus, this investigation confirms the results of the previous study. It is assumed that the laser pulse energy of the investigated U-LSP process is not high enough for the treatment of X5CrNiCu15-5 to induce significant plastic deformation and compressive residual stresses at a feed rate of V = 20 m/min. Tensile residual stresses after U-LSP with V = 10 m/min may be explained thermal effects during the process. In this case, it is assumed that the laser pulses lead to a local heating of the surface without the ablation layer. The heating and cooling during the process lead to tensile residual stresses. The ablation layer that reduces thermal effects on the surface condition after LSP was not used in this test series. Test series with similar parameters but with the ablation layer seem to minimize the thermal effects and lead to compressive residual stresses at the surface. However, these compressive residual stresses are still significantly lower than the induced compressive residual stresses via SP and C-LSP. It is assumed that the laser pulse energy of the U-LSP process is not high enough to induce significant plastic deformation in the surface layer. For AlZnMgCu1.5, similar residual stress states were reached with U-LSP according to C-LSP; however, with a treatment time that was between 20 and 100 times lower (see area rate at Table 4). For the surface treatment of this Al-alloy U-LSP with the current laser pulse energy, a 0.001 J/pulse and a repetition rate of 400 kHz seem to be an economic alternative to conventional LSP processes.

The following conclusions are made:

Significant lower roughness was determined after C-LSP and U-LSP compared to SP. U-LSP roughness values of $R_{a}$ < 0.2 µm and $R_{a}$ < 1.5 µm could be reached that is below the roughness after C-LSP or milling (DC).

Higher compressive residual stresses at the surface were determined after SP and C-LSP compared to U-LSP. For AlZnMgCu1.5, a similar residual stress level between U-LSP and C-LSP could be reached (around -200 MPa at the surface). For X5CrNiCu15-5, the intensity of the U-LSP process was not high enough to induce significant compressive residual stresses.

The highest effect of the U-LSP parameters on the residual stress state has the confining layer. However, the best results were reached with zinc, black paint, or even without a layer. In this investigation, an additional layer for the U-LSP treatment does not seem necessary.

The penetration of the compressive residual stresses after U-LSP was around 200 µm and significantly below the penetration that was reached with C-LSP or SP.

A change in the phase composition (a higher portion of oxide phases and a lower portion of the martensite phase) was determined after the C-LSP process but not after SP.

In general, for the specimens in this study made of AlZnMgCu1.5, similar or lower roughness values were determined for U-LSP compared to other surface treatment processes while slightly lower compressive residual stresses were determined; however, with a lower penetration depth. The shorter treatment times of U-LSP (ca. 10 s in this case at 35 × 35 mmm^2^ compared to C-LSP of 95 s) may make this process attractive if equivalent lasers are available for industrial application and if the investment costs for U-LSP and C-LSP equipment is similar. For X5CrNiCu15-5, the laser pulse energy does not seem high enough to induce significant plastic deformation or residual stresses in the surface layer. Further developments of the Fraunhofer CAPS-Cluster may address this issue and allow the mechanical surface treatment of materials with a higher yield strength via U-LSP in the future.

## Figures and Tables

**Table 1:** Chemical composition of the investigated materials.

(Fe/Al Balanced) Composition [%]	C	Si	Mn	Ni	Cr	Cu	Mo	Nb	P	S
X5CrNiCu15-5	0.028	0.31	0.52	5.11	14.96	3.36	0.2	0.285	0.019	<0.001
	Fe	Si	Mn	Ni	Cr	Cu	Mg	Zn	Ti	Si
AlZnMgCu1,5	0.17	0.075	0.05	0.016	0.18	1.5	2.4	5.7	0.02	0.075

**Table 2:** Material properties of the investigated materials (acc. to certificate).

	Yield Strength [MPa]	Tensile Strength [MPa]	Elongation [%]
X5CrNiCu15-5	min 1000	min 1070	min 11
AlZnMgCu1-5	min 480	min 540	min 7

**Table 3:** Constant process parameters for the conventional Laser Shock Peening (C-LSP) and ultrafast Laser Shock Peening (U-LSP).

	Pulse Duration [ns]	Repetition Rate [Hz]	Laser Pulse Energy [J]	Focus Diameter d [mm]	Offset c [mm]
C-LSP	10	10	2	1.3	1.0
U-LSP	0.001	400,000	0.001	0.06	0.025–0.1

**Table 4:** Variable process parameters for the conventional Laser Shock Peening (C-LSP) and ultrafast Laser Shock Peening (U-LSP).

	Feed Rate V [mm/s]	Defocusing Distance b [mm]	Offset c [mm]	Number of Shots [-]	Coverage [%] *	Area Rate [mm^2^/s]
C-LSP				1	103	12.9
	12.9	0	1	2	205	6.5
				4	411	3.2
U-LSP	20,000		0.1		57	2000
		0	0.05	1	113	1000
			0.025		226	500
			0.1		481	2000
		6	0.05	1	962	1000
			0.025		1924	500
			0.1		905	2000
		10	0.05	1	1810	1000
			0.025		3619	500
			0.1		226	500
		0	0.05	4	452	250
			0.025		905	125
			0.1		57	2000
	10,000	0	0.05	4	905	125
			0.025		1810	62.5

* calculated according to overlap of the spot or focus diameter d of subsequent laser pulses.

**Table 5:** Measurement parameters for the determination of residual stresses at both jackets.

Material	Method	Radiation	Lattice Plane	Kollimator ∅	Exposure Time	Distance L	$\mathrm{Tilt}\;\mathrm{Angle}\;\psi_{0}$
		[-]	[-]	[mm]	[s]	[mm]	[°]
X5CrNiCu15-5	sinψ^2^	CrKβ	{211}	2	1360	-	−45 to 45 (12×)
	cosα				16	51	35
AlZnMgCu1.5	sinψ^2^	CuKα	{422}		3225	-	−45 to 45 (12×)
	cosα				15	37	25

## Data Availability

The data presented in this study are available on request from the corresponding author. The data are not publicly available because it also forms part of an ongoing study.
